# Vibro-Shock Dynamics Analysis of a Tandem Low Frequency Resonator—High Frequency Piezoelectric Energy Harvester

**DOI:** 10.3390/s17050970

**Published:** 2017-04-27

**Authors:** Darius Žižys, Rimvydas Gaidys, Vytautas Ostaševičius, Birutė Narijauskaitė

**Affiliations:** 1 Faculty of Mechanical Engineering and Design, Kaunas University of Technology, Studentu 56, Kaunas LT-51368, Lithuania; rimvydas.gaidys@ktu.lt; 2 Institute of Mechatronics, Kaunas University of Technology, Studentu 56-123, Kaunas LT-51368, Lithuania; vytautas.ostasevicius@ktu.lt; 3 Faculty of Mathematics and Natural Sciences, Kaunas University of Technology, Studentu 50, Kaunas LT-51368, Lithuania; birute.narijauskaite@ktu.lt

**Keywords:** vibration energy harvesting, vibro-shock piezoelectrics, multi-beam dynamics, frequency up-conversion, numerical modeling

## Abstract

Frequency up-conversion is a promising technique for energy harvesting in low frequency environments. In this approach, abundantly available environmental motion energy is absorbed by a Low Frequency Resonator (LFR) which transfers it to a high frequency Piezoelectric Vibration Energy Harvester (PVEH) via impact or magnetic coupling. As a result, a decaying alternating output signal is produced, that can later be collected using a battery or be transferred directly to the electric load. The paper reports an impact-coupled frequency up-converting tandem setup with different LFR to PVEH natural frequency ratios and varying contact point location along the length of the harvester. RMS power output of different frequency up-converting tandems with optimal resistive values was found from the transient analysis revealing a strong relation between power output and LFR-PVEH natural frequency ratio as well as impact point location. Simulations revealed that higher power output is obtained from a higher natural frequency ratio between LFR and PVEH, an increase of power output by one order of magnitude for a doubled natural frequency ratio and up to 150% difference in power output from different impact point locations. The theoretical results were experimentally verified.

## 1. Introduction

In recent decades smart gadgets, various micro electro-mechanic devices, and other MEMs have become popular. For such devices, the problem of energy supply is becoming increasingly important. Usually it is difficult or nearly impossible to supply the device with an external energy source, and batteries tend to run out quickly, thus, researchers have started to develop energy harvesters that utilize ambient energy sources (solar, mechanical vibrations, thermal gradients, etc.) to meet the changing power supply demands [1] since it was recognized that the power consumption goal for devices of size <1 cm^3^ is 100 µW, as described by [2]. MEMS, wireless sensor nodes, micro-RF receivers and other similar devices all use power ranging from 10 nW to 1 mW, which is acknowledged as a realistic goal for modern energy harvesters. The mechanical energy domain [3] is of the utmost interest for this purpose since mechanical energy is an inexhaustible, promising, and abundant source of environmental motion energy [3,4,5], especially in industrial and mechanically driven environments. Vibration energy was identified by several authors as the easiest energy source to harvest [3,5,6]. An absolute majority of investigated energy harvesting devices in the field utilize one of three main transduction mechanisms: piezoelectric, electrostatic and electromagnetic. Wang et al. [7] developed an electrostatic vibration energy harvester consisting of a four wafer stack. The 1 cm^3^ volume device operated at 179.5 Hz frequency and produced 32.5 nW power output at 0.03 g acceleration. Wang et al. have further improved their design [8]. The new device operated at a higher acceleration of 1 g and an excitation frequency of 96 Hz, and its power output was increased up to 0.15 µW at a cost of volume increase up to 1.43 cm^3^. Zhang et al. [9] proposed a broadband electrostatic energy harvester. Broadband input was achieved by utilizing a dual resonant structure, whereby one resonator is at resonance and at large oscillation amplitudes so a collision between the two resonators occurs further increasing the power output. The device was proven to be operational in the 36.3–48.3 Hz frequency band producing up to 6.2–9.8 µW of power output at 9.3 m/s^2^ vibration amplitude. To solve the gap-spacing control problem and sticking of top-to-bottom structures in electrostatic in-plane devices Suzuki et al. have proposed a novel method in their initial work [10] and its further continuation [11]. The gap was passively controlled by electrostatic repulsive forces which are created using a patterned electret. The drawback of this is that the electret needs initial charges on the electrodes thus a comparatively low power output of 1 µW at 2 g and 63 Hz was achieved, although the results were further improved in [11].

As for the electromagnetic transduction mechanism, Yang et al. [12] have developed a non-resonant electromagnetic wideband device for energy harvesting from low frequency sources. The device consists of a magnet placed in a PCB-copper coil tube. The device demonstrated a frequency range of 40–80 Hz and produced a maximum power output of 0.4 µW at 50 Ω load resistance. Another work done in field of electromagnetic transduction mechanism was done by Sardini et al. [13] and involved a nonlinear electromagnetic generator with planar resonators. By choosing polymeric materials for their nonlinear resonators the authors managed to lower the device’s operation frequency from 100 Hz to 30–40 Hz producing a maximum instantaneous power and voltage of 153 µW and 378 mV. It is needed to note the power output here is not an rms but rather a peak value.

Some papers have also presented hybrid energy harvesting devices where two or more transduction mechanisms are utilized for energy harvesting in the same device. An example of such a device could be the harvester developed by Mahmoudi et al. [14]. There a hybrid nonlinear vibration harvester based on piezoelectric and electromagnetic transduction is described. The device consisted of a bimorphic piezoelectric cantilever and a magnet attached to the center of the piezoelectric beam and placed between two fixed magnets and a copper coil. The authors demonstrated a 60% power density increase (932 µW·cm^−3^·g^−1^) and 29% increase in frequency bandwidth (153–198 Hz) if compared to previous devices with pure magnetic levitation. The piezoelectric mechanism accounted for 39% of total power output, while the electromagnetic one provided 61%. It is also necessary to mention that this improvement is only possible if the device is excited beyond its critical Duffing amplitude [15,16].

Abed et al. [17] demonstrated a purely electromagnetic transducer—multimodal vibration energy harvester consisting of arrays of coupled levitated magnets. Using a multi-objective optimization technique, nonlinearly coupled 2-DOF and 3-DOF vibration energy harvesters (VEH) have been investigated proving their superiority over uncoupled VEHs. A 2-DOF VEH working at the 5.1–12 Hz frequency band demonstrated a power output density of 10.4 µW·cm^−3^·g^−2^ while a 3-DOF VEH demonstrated a 4.6–14.5 HZ frequency range and 20.2 µW·cm^−3^·g^−2^ power output density. A similar device was investigated by the same authors [18], but this time with two central magnets instead of one. This nonlinear multi-modal VEH with magnetically coupled beams was investigated theoretically and at an operating frequency range of 43–80.5 Hz a 60 µW·cm^−3^·g^−2^ power density was demonstrated.

The piezoelectric transduction mechanism is recognized as one of the most effective for vibration energy harvesting. In typical applications, the transducer is mounted on a vibrating structure to create a direct piezoelectric effect for energy harvesting. Various authors have investigated this setup and different results have been obtained. Reference [19] reported a “sea weed” inspired harvester built of foam and PVDF layers which harvests energy from a turbulent flow of liquid. Modeling results have shown an average power output of 850 µW at 10 Hz vibration frequency.

Different approaches have been taken to the piezoelectric transduction mechanism, the simplest type of which is a piezoelectric cantilever as investigated by [20]. Reference [21] obtained ~50 µW from a rectangular cantilever piezoelectric harvester operating at ~27 Hz base excitation frequency. The piezoelectric transduction mechanism usually consists of a cantilever and a layer or layers of piezoelectric material that is bonded on its surfaces, and a unimorph cantilever-type piezoelectric harvester with PZT thick films was demonstrated by [22]. A maximum electrical power of 17.3 mW was obtained at 20 Hz at a comparatively high acceleration of 4 g. Reference [23] investigated a 50 mm long, 30 mm wide bimorphic piezoelectric energy harvester made of brass and PZT-5H with four different 12 g proof mass configurations at 1 g acceleration and 48 Hz base excitation; the authors obtained a power output of 18.47 mW. In the same work [23], a piezoelectric bimorph with a similar 9.8 g proof mass but four different geometries was mounted on a 21.85 mm long, 3.2 mm wide cantilever made of brass with two PZT-5H layers mounted on top and bottom. Up-frequency sweeping at 0.5 g acceleration revealed that the maximum power output obtained was 1 mW at ~20 Hz base excitation frequency (the proof mass height-width ratio was 3:1) and 15 µW at 170 Hz (when the proof mass height-width ratio was 5:1), thus demonstrating the dependency of the harvested power on the natural and excitation frequencies of the devices. Similar devices were also investigated by [23,24,25].

For maximization of the power output of such a device, the natural frequency of the transducer has to be matched to the vibration frequency of the structure. A slight drift from resonance significantly lowers the Piezoelectric Vibration Energy Harvester (PVEH) energy output, which results in a serious drawback, i.e., it has a narrow bandwidth, and comparatively high frequencies are necessary for high power density generation. In other words, the challenge lies with the spectrum of natural vibrations. The energy of natural motion energy spreads over low frequencies and its variation from source to source and even from time to time at the same source cannot be predicted. The problem of a narrow operation frequency bandwidth has been tackled by nonlinear multimodal wideband PVEHs. Reference [26] used a nonlinear wideband multimode energy harvester with three masses and achieved an impressive resonant operation bandwidth of 20 Hz (from 105 to 125 Hz) with a peak-to-peak voltage output of 32 V during up-sweep and 0.8 g excitation. Research work [27] developed a micro-energy harvester operating at sub-100 Hz frequencies. The silicon shim was covered with aluminum nitride (AlN) energy harvesting elements, and a chip was used as a proof mass. The first three natural frequencies of 71 Hz, 84 Hz, and 188 Hz were measured experimentally, and an operation bandwidth of 10 Hz was obtained for the second mode shape under a base excitation of 0.2 g. A maximum open circuit voltage of 1 V and power output of 136 nW with load resistance of 2 MΩ have been achieved. With an advanced power conditioning circuitry and A1N element replaced with PZT, a power output of ~3.1 µW is predicted.

Reference [28] investigated the feasibility of exploiting the second vibration mode and nonlinear oscillations to widen the operation bandwidth of the rectangular piezoelectric bi-stable laminate for broadband vibration energy harvesting at relative higher frequencies, but with relative low excitation acceleration. A proof mass of 15 g was added closer to the fixed end of the cantilever (150 mm × 50 mm) to further lower the natural frequency from 99 Hz to 65 Hz in the case of a relatively small excitation acceleration of 0.4 g. The achieved operation band frequency was 11 Hz with an open circuit voltage output of 23 V at a lower end of the band and 36 V at the upper end. Using the second vibration mode also helped to tackle the second problem, i.e., relatively high vibration amplitudes at low frequencies. At low frequencies (up to 50 Hz) cantilevers vibrate with vigorous amplitudes of up to several centimeters, even at low accelerations (~1 g). This problem was also tackled in [29], where a low-frequency meandering piezoelectric vibration energy harvester was investigated. The measured power output and normalized power density were 118 μW and 5.02 µW·mm^−3^·g^−2^, respectively, when excited by an acceleration magnitude of 0.2 g at 49.7 Hz. Different authors also investigated the possibility to use advanced power conditioning circuits [30,31] to increase the power density of such devices. One way to reduce the vibration amplitude is to increase the mechanical damping factor but that way the energy flow to the device decreases, and the major part of the energy flowing into the device will still be consumed by the mechanical damping element further decreasing the part of the energy available for energy harvesting via electromechanical coupling [32]. The energy density of transducers operating at low frequency is very low due to the size of the transducers themselves, as described by [18]. Shanshan et al. [33] reported a bi-resonant structure consisting of two cantilevered energy harvesters with attached PVDF films and resonant frequencies of 15 and 22 Hz. As one of two cantilevers reached its peak vibrations at resonant vibration frequencies dynamic contact occurred further increasing the power output. The experiments revealed that at acceleration of 1 g the bandwidth of the device was 14 Hz (14–28 Hz) and peak power output was 0.35 µW. The same authors have investigated another dual resonator system harvesting energy from both forced vibration and impact coupling [34]. Both experiments and modelling proved the dual resonator system produced higher energy output than both cantilevers separately. The device produced its highest power output at a center frequency of 20 Hz and 25 Hz bandwidth producing 50 µW rms power output under a rms acceleration amplitude of 6.3 m/s^2^ (the two cantilevers separately produced 18 μW for device 1 and 25 μW for device 2. The described nonlinear multimodal wideband PVEHs also include mono/bi/multi-stable structures, frequency up-converters, and generators with active or passive resonance tuning. In frequency up-conversion, the abundant environmental motion energy is absorbed by a low frequency (LF) resonator which transfers it to the high frequency PVEH via impact coupling, and a decaying output signal is produced. The energy is then transferred from the mechanical to the electrical domain via an electromechanical conversion mechanism. Several authors have investigated energy transfer from LF resonators to high frequency PVEHs via magnetic coupling, Tang et al. [35] have proposed a miniature piezoelectric vibration energy harvester with a frequency up-conversion which is achieved through magnetic repulsion forces. The main advantage of this type of device is that the frequency up-conversion is non-contact. The power output achieved was 10 µW at 1 g acceleration and a frequency range of 10–22 Hz. A similar device was also investigated by [36]; the drawback of such devices is that the ferromagnetic elements further increase the volume of the device thus decreasing the output energy density. The device described in [37] achieved a bandwidth of 8 Hz (from 10 to 18 Hz). At 10 Hz excitations with a peak acceleration of 1 g, the harvester responds at a higher frequency of 20 Hz and gives a peak power of 2.68 mW and a peak-to-peak voltage of 2.62 V across a load of 220 Ω. The average power density of 65.74 µW·m^3^ obtained at 10 Hz 1 g excitations monotonically increases with frequency up to 341.86 µW·cm^3^ at 18 Hz. Another study [38] presented an electromagnetic device operating in sub-100 Hz frequencies by means of EM induction which was accomplished by installing a coil and a magnet on two resonating cantilevers. A macroscale device demonstrated a 170 nW maximum power and 6 mV maximum voltage and for a MEMS version the power and the maximum voltage from a single cantilever are 3.97 µW and 76 mV, respectively, under vacuum conditions. Frequency up-conversion with impact coupling has also been investigated by several authors. Reference [39] described an frequency up-converting wideband vibration energy harvester with impact coupling. At low frequency base excitation of 18 Hz, it induced 374 Hz free vibrations in PVEH via the impact coupling. The device achieved a 4 Hz bandwidth (18 Hz to 22 Hz) and 0.33 µW power output at 0.5 g acceleration. Research work [40] presented an extra-low-frequency-driven (almost human motion) frequency up-conversion signal, where the device managed to achieve a conversion ratio of 12.5 from excitation of 4 Hz. The displacement was limited to 2.2 mm.

From the literature review, it can be seen that the impact-driven vibration energy harvesters have the highest potential in the systems with low or uneven base excitation frequencies since the power is generated not by a low frequency resonator, which can as well be a free mass, but by a piezoelectric harvester, in which the vibrations are induced via impact or magnetic coupling. Such low frequency and high acceleration environments can be found in human motion. Vibrations induced in PVEH via impact coupling from a low frequency resonator or another impacting body are of higher frequency, and higher vibration frequencies are known to carry more energy. During the impact, higher vibration modes are also produced; thus, choosing the best contact point location plays an essential role. PVEH’s natural frequency to external excitation frequency ratio is also an important aspect to ensure the maximal power output from such a system. This paper concentrates on the investigation of the impact of these parameters on the power output of PVEHs. A hypothesis that higher power output can be achieved if higher vibration modes are induced by locating the point of impact at the strain node of the second vibration mode is verified. It is also intended to investigate the influence of excitation to the first natural frequency of the PVEH ratio on the power output of the harvester.

## 2. Modeling of LFR and PVEH Vibro-Shock Harvesters

**Figure 1:**
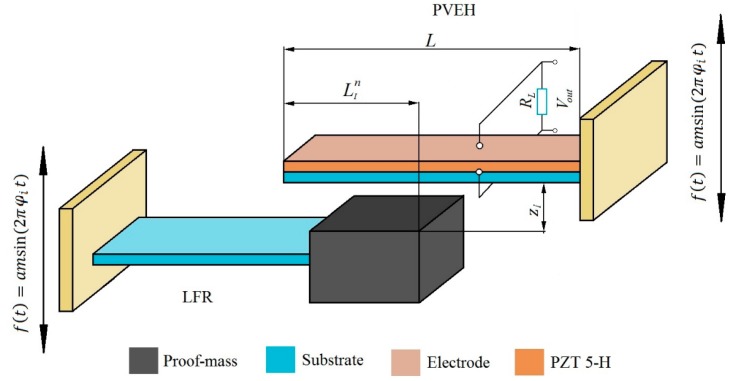
Schematic representation of harvester tandem.

A frequency up-converting tandem is investigated. The tandem consists of a low frequency resonator (LFR) and a high frequency piezoelectric vibration energy harvester (PVEH). A schematic representation of the energy harvesting “tandem” can be seen in Figure 1. The LFR consists of a steel cantilever and a proof mass attached to its tip. Different transverse vibration eigenfrequency ratios were achieved by varying the geometry and proof-mass of LFR while the geometry of PVEH was kept constant for the entire analysis. The piezoelectric cantilever was modeled as a uniform composite beam subjected to linearly elastic deformations and geometrically small oscillations with reference to the Euler–Bernoulli beam assumption. Table 1 presents the mechanical and geometrical of properties of the PVEH. A thin layer of PZT-5H piezoelectric material was bonded on the upper surface of the generator. The upper and lower surface of the piezoelectric material layer was covered with ideally conductive electrode layers of negligible thickness, the function of which is to create a uniform potential.

Several different energy harvesting tandem geometries were investigated with different LFR to PVEH transverse vibration eigenfrequency ratios for frequency up-conversion. Five different configurations of LFR were investigated, with first transversal mode vibration eigenfrequencies being $\omega_{1}^{LFR}$ = (311, 207, 155, 103, 77) Hz, where *i =* 1, 5. 

The harvester tandem is actuated kinematically in a vertical direction by the harmonic law of motion with frequency $\varphi_{i}$ equal to corresponding $\omega_{1}^{LFR}$. The high frequency PVEH is suspended above LFR at distance $z_{gap}$, where $z_{gap}$ is the distance between LFR and PVEH. As the low frequency resonator, transverse vibration amplitude reaches $z_{gap}$ value (PVEH edge) due to kinematic actuation, a dynamic point contact occurs between LFR and PVEH creating a vibro-shock system.

The tandem’s contact point $L_{I}^{n}$ (where *n* = $\bar{0.6}$) between LFR and PVEH was investigated by shifting the contact point position from the tip 0 L of the piezoelectric cantilever towards its fixed end with a step of 0.1 L up to 0.6 L, where L is the length of PVEH, (1–0 L, 2–0,1 L, 3–0.2 L, 4–0.3 L, 5–0.4 L, 6–0.5 L, 7–0.6 L) and *n* = 0 corresponds to the tip 0 L of the cantilever and *n* = 7 to 0.6 L distance from the tip of PVEH. The geometric and piezoelectric properties of the piezoelectric material (PZT-5H) layer are listed in Table 2.

An electric circuit consisting of electric load resistance $R_{l}$ and a voltage generating piezoelectric element was attached to the PVEH model. An electrical load allows predicting the amount of power generated by the device. This is a simplified approach to an energy harvesting circuit since in the real world it is more complicated than a simple resistive load. In this paper, however, the authors did not focus on the electrical properties of such a device.

A coupled 2D piezoelectric-circuit finite element model (CPC-FEM) was created to solve this problem. The system was modeled using COMSOL multi-physics software with SPICE piezoelectric circuit attached. Modeling was performed using Lagrange-quadratic finite elements for plane-strain approximation. The LFR and PVEH tandem was excited cinematically by the harmonic law in the transversal direction; a kinematic effect in the model is described as a body load with magnitude controlled by imposed acceleration (a = 0.85 g) and excitation frequency $\varphi_{i}$ (where $\varphi_{i}$ = $\omega_{1i}^{LFR}$). The transverse vibration eigenfrequency ratios investigated were $\omega_{1}^{PVEH}$/$\omega_{1}^{LFR}$ = (2; 3; 4; 6; 8), where $\omega_{1}^{PVEH}$ and $\omega_{1}^{LFR}$ are the first transverse vibration eigenfrequencies of the high frequency PVEH and LFR, respectively.

### 2.1. Constitutive Equations

The proposed frequency up-converting tandem was modeled as a spring-mass-damper system as described by various authors [41,42,43], consisting of two spring-damper systems (namely the LFR and high frequency PVEH). Both cantilevers are driven by cinematically actuated harmonic frequency excitation corresponding to the LFR’s first natural transversal vibration mode frequency. It should be noted that harmonic base excitation accounts only for the part of PVEHs’ energy input, while the other part is supplied by the dynamic vibro-shock contact between the LFR and PVEH.

The direct piezoelectric effect is used for energy harvesting. To polarize a poled material, a mechanical strain has to be applied. As a result, a fixed electrical charge is induced on the surface of this material. By having that surface sandwiched between a pair of electrodes, we can collect these charges. The density of the induced charge is linearly proportional to the strain in the material, and thus proportional to the externally applied stress. This relationship can be described mathematically as follows:

(1) $$P_{pe}=d\;\times \;T$$

where $P_{pe}$ is the piezoelectric polarization vector with magnitude equal to the fixed charge density obtained due to the direct piezoelectric effect, *d* is the piezoelectric strain, and the coefficient *T* is the stress affecting the piezoelectric material. The subscript “*pe*” means that the value is generated by a piezoelectric effect, and externally applied values have no subscripts.

If the elastic properties of the material are taken into account, Equation (1) can be rewritten as shown in Equation (2):

(2) $$P_{pe}=d\;\times \;T\;=\;d\;\times \;p\;\times \;S\;=\;e\;\times \;S$$

where *p* is the elastic constant relating the generated stress and the applied strain, *e* is the piezoelectric stress constant, and *S* is the applied mechanical strain. The side effect of piezoelectric effect is that it increases the stiffness of the material and contributes to dielectric constants, which is described in detail in [44].

The piezoelectricity is a cross-coupling between the elastic variables (stress *T* and strain *S*) and the dielectric variables (elastic charge density *D* and electric field *E*). In the linear theory of piezoelectricity [44], the tensor relation to identify the coupling between mechanical stress, mechanical strain, electric field, and electric displacement is given by:

(3) $$S_{p}=s_{pq}^{E}T_{q}\;+\;d_{pk}E_{k}$$

(4) $$D_{i}\;=\;d_{iq}T_{q}+\varepsilon_{ik}^{T}E_{k}$$

where $s_{pq}^{E}$ is the elastic compliance tensor at constant electric field, $\varepsilon_{ik}^{T}$ is the dielectric constant tensor under constant stress, $d_{pk}$ is the piezoelectric constant tensor, $S_{p}$ is the mechanical strain in the p direction, $D_{i}$ is the electric displacement in the i direction, $T_{q}$ is the mechanical stress in the *q* direction, and $E_{k}$ is the electric field in the *k* direction. The mathematical formulation of the piezoelectric equations for the finite element method could be expressed by the following system of differential equations:

(5) $$M\ddot{z}+C_{zz}\dot{z}+K_{zz}z+K_{z\Phi }\Phi =F$$

(6) $$K_{z\Phi }^{t}z+K_{\Phi \Phi }\Phi =Q$$

where $\ddot{z}$ is a nodal point acceleration vector, $\dot{z}$ is a nodal point velocity vector, and z is a nodal point displacement vector; Φ is the electrostatic potential and is a scalar, the subscript *z* refers to mechanical quantities, whereas the subscript Φ refers to electrical ones, the combination of the two refers to electromechanical coupling matrices; *M*, *C* and *K* are global matrices and *z*, *F*, Φ and *Q* denote vectors. Equations (5) and (6) could be expressed in the matrix form:

(7) $$\left[\begin{array}{cc} M_{zz} & 0\\ 0 & 0 \end{array}\right]\left(\begin{array}{c} \ddot{z}\\ \ddot{\varphi } \end{array}\right)+\left[\begin{array}{cc} C_{zz} & C_{z\varphi }^{t}\\ C_{z\varphi } & C_{\varphi \varphi } \end{array}\right]\left(\begin{array}{c} \dot{z}\\ \dot{\varphi } \end{array}\right)+\left[\begin{array}{cc} K_{zz} & K_{z\varphi }^{t}\\ K_{z\varphi } & K_{\varphi \varphi } \end{array}\right]\left(\begin{array}{c} z\\ \varphi \end{array}\right)=\left(\begin{array}{c} F\\ Q \end{array}\right)$$

A nonlinear viscoelastic contact model by Hunt and Crossley [43] was employed to achieve a mechanical contact between LFR and the piezoelectric generator. The model is used in cases involving a small contact surface and is valid for direct central and frictionless contact. The dynamic contact between LFR and PVEH is expressed by the spring constant $k_{c}$ and damping factor $c_{c}$. The gap between LFR and PVEH is given by *z_gap_* While the gap (*z_gap_* > 0) $k_{c}$ and $c_{c}$ have no effect on the system, only the spring-damper systems of both cantilevers are in function. The external mechanical load vector *F* describes the mechanical excitation of the LFR and PVEH and could be expressed as follows:

(8) $$\left(F\right)=\{\begin{array}{cc} \left(F_{k}\right), & p_{ls}\left({\dot{z}}_{ls},\;z_{ls},\;t\right)\geq 0\\ \left(F_{k}\right)+\left(P_{c}\left(\dot{z},z,t\right)\right), & p_{ls}\left({\dot{z}}_{ls},\;z_{ls},\;t\right)<0 \end{array}$$

where (*F_k_*) is the kinematic excitation force, $\left(P_{c}\left(\dot{z},z,t\right)\right)$ is a nonlinear interaction vector in the contact pair, $p_{ls}$ is the contact pair nonlinear interaction force at the contact point of the PVEH.

During the contact, LRF and PVEH systems function in parallel, and the contact force is described by Equation (9). The model is described in more detail in [44]:

(9) $$P_{C}=k_{c}z^{\alpha }+c_{c}z^{\alpha }\dot{z},\;\;\mathrm{for}\;z<0\;\mathrm{and}\;\dot{z}<0$$

where $k_{c}$ is a contact stiffness coefficient, $c_{c}$ is a contact damping coefficient, α is a force exponent depending on contact surface geometry (*α* = 2 is assumed). This system of equations could be solved by direct numerical integration methods.

### 2.2. Analysis of the Energy Harvesting of PVEH under Vibro-Shock Excitation

**Figure 2:**
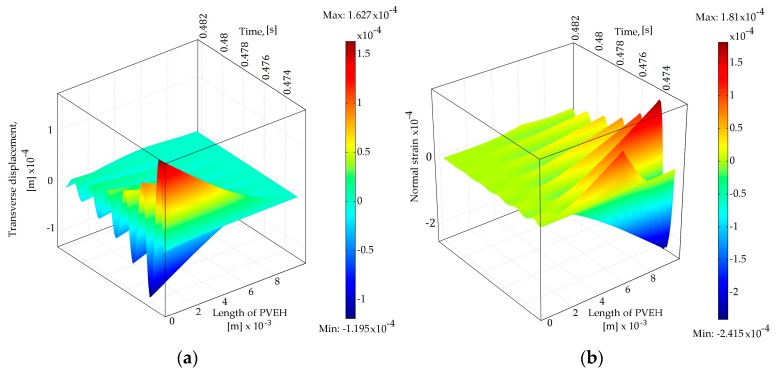
Dynamic process of the PVEH from the instant of impact to fully decayed vibration (LFR—103 Hz, contact point position at 0.6 L) (**a**) transverse displacement and (**b**) normal strain distribution through the length of the PVEH.

The PVEH is driven by the energy from the dynamic impact occurring between the LFR and PVEH during base excitation of the harvesting tandem. The nonlinear dynamic behavior is controlled by the equation of motion, which is solved to find the dynamic response. The dynamic analysis of different LFR geometries was performed to model the PVEH dynamics process, then the LFR was kinematically actuated by its first transverse vibration mode eigenfrequency. A transient analysis was conducted to obtain the dynamic response of the LFR-PVEH tandem under harmonic base excitation and dynamic contact between the two cantilevers under open circuit conditions (*R_L_* → ∞), as shown in Figure 2.

**Figure 3:**
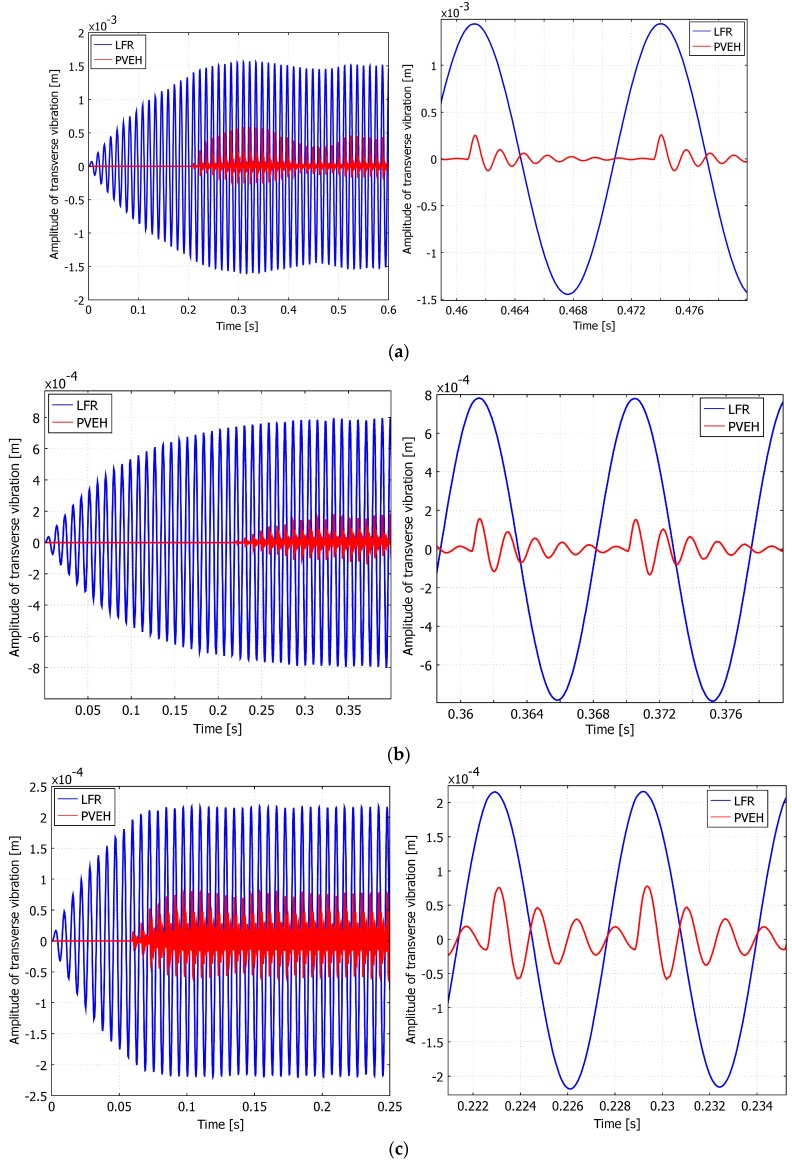

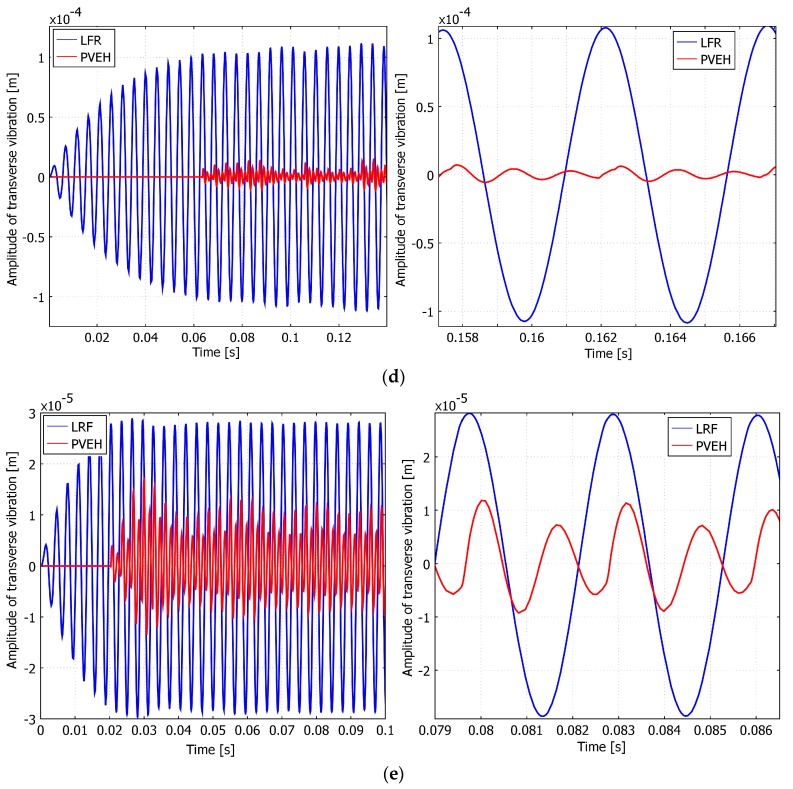
Transverse vibrations of LFR (blue) and high frequency PVEH (red) tip point. Contact position $L_{I}^{n}$ = 0.5 L of PVEH and natural frequency ratio $\omega_{1}^{PVEH}/\omega_{1}^{LFR}=$ (**a**) 8; (**b**) 6; (**c**) 4; (**d**) 3; (**e**) 2.

From Figure 2a,b it can be seen that the amount of normal strain is highest at the fixed end of the cantilever (Figure 2b) when the displacement is at its maximum as seen in Figure 2a. As the vibration decays, the normal strain in the length of PVEH also decays. From Figure 2b it can also be seen that the amount of positive normal strain is higher than that of negative one, which can be related to the impact since LFR approaches and impacts PVEH from the bottom side. In Figure 3 the dynamic responses of different energy harvesting tandem configurations (LFR $\omega_{1}^{LFR}$ = (311, 207, 155, 103, 77 Hz) resulting in $\omega_{1}^{PVEH}$/$\omega_{1}^{LFR}\;$= (2, 3, 4, 6, 8) respectively) are presented, and then the contact point is located at 0.5 L of PVEH.

From these figures, it can be seen that the vibration amplitude “pulsates” since the “change-over” between kinetic energy is taking place between the cantilevers. It can also be seen from these figures that the lower frequency of the LFR, the longer it takes to reach the steady state. One could also predict repetitive strikes between the PVEH and the LFR after the impact due to the large differences in the natural frequencies. This can be observed very well in Figure 3a, where in the transverse response of the PVEH, after the impact of LFR, the peak of the amplitude is split into two, i.e., a clear result of another impact right after the first one. In the next section the results obtained from modeling are processed using numerical methods.

## 3. Numerical Analysis of PVEH Dynamics Process and Power Output

**Figure 4:**
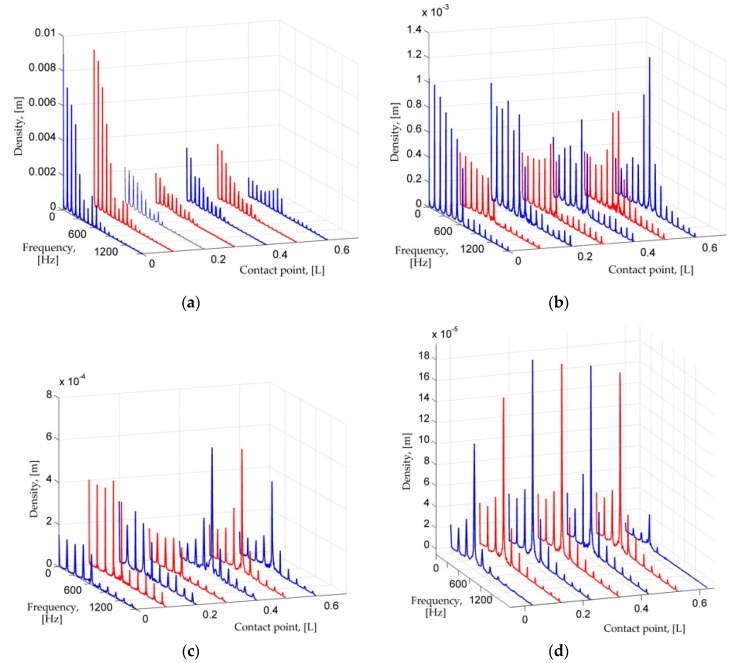

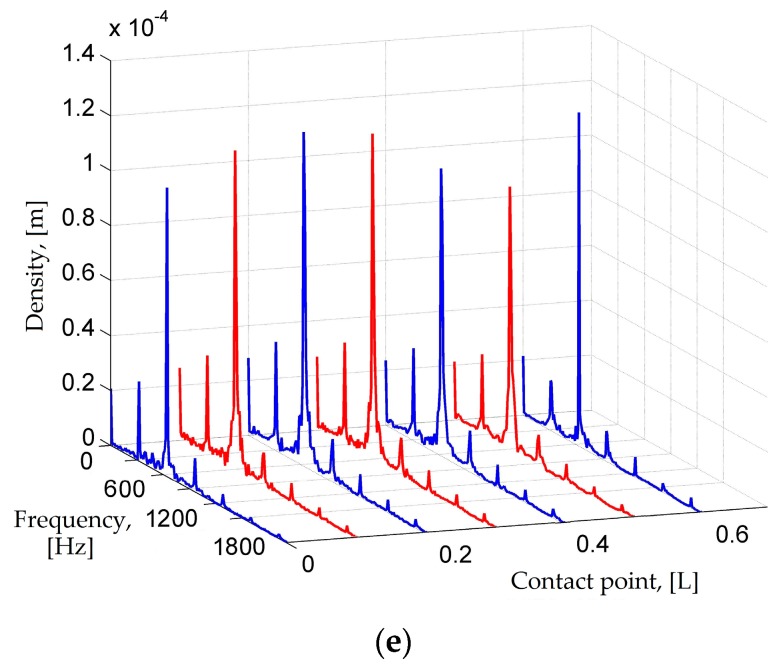
DFT of LFR-PVEH tandem at different dynamic contact points and natural frequency ratio $\omega_{1}^{PVEH}/\omega_{1}^{LFR}=$ (**a**) 8; (**b**) 6; (**c**) 4; (**d**) 3; (**e**) 2.

In the numerical analysis, the results obtained from the transient analysis were analyzed and processed using the MATLAB software. In Figure 3a–e it can be clearly seen that the transverse vibrations of the PVEH under impact excitation are not entirely sinusoidal, but rather pulsating. Discrete Fourier transform analysis or DFT has been done to further investigate these signals. One can predict that the response of PVEH shall be a superposition of both the excitation frequency of LFR and the response of PVEH which is predicted to be its first transverse vibration eigenfrequency (622 Hz). The obtained frequency spectral density can be seen in Figure 4a–e. It is obvious that at a large natural frequency ratio $(\omega_{1}^{PVEH}$/$\omega_{1}^{LFR}=8)$ there is a broad spectrum of dominant frequency density spikes, and the number of spikes tends to decrease as the point of dynamic contact goes further from the tip (0 L) of the cantilever to the fixed end of PVEH (0.6 L). There the density of the first natural frequency of PVEH $\omega_{1}^{PVEH}$ is close to that of $\omega_{1}^{LFR}$ with some iteratives of $\omega_{1}^{PVEH}$ in between. These iteratives represent the repetitive bouncing contact of PVEH to LFR during the approach stage of both cantilevers. The lower frequency ratio is, the less noise there is in the DFT spectrum, and the more expressed the fundamental frequencies of the investigated cantilevers are. This is because the lower the ratio is, the less bouncing contact can be expected.

Further, the electrical properties of PVEH are investigated. Since the piezoelectric element produced the AC energy output, the root mean square values were calculated for each characteristic curve. The formula used for this purpose is shown in Equation (10):

(10) $$\mathrm{RMS}=\;\sqrt{\frac{\sum_{i=0}^{n}a_{i}^{2}}{n}}$$

where *a* is an array of elements or points in the obtained electrical response curves and *n* is the number of these elements.

**Figure 5:**
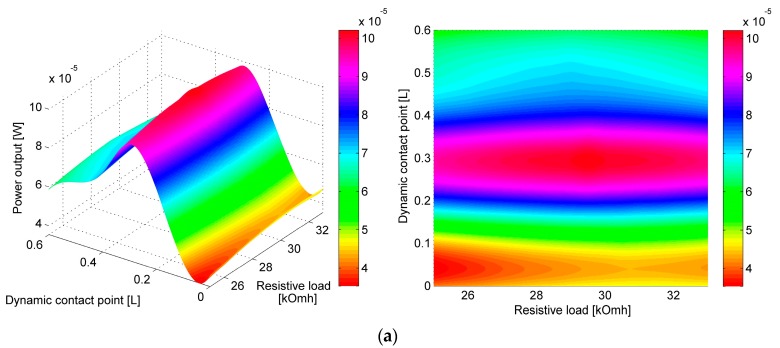

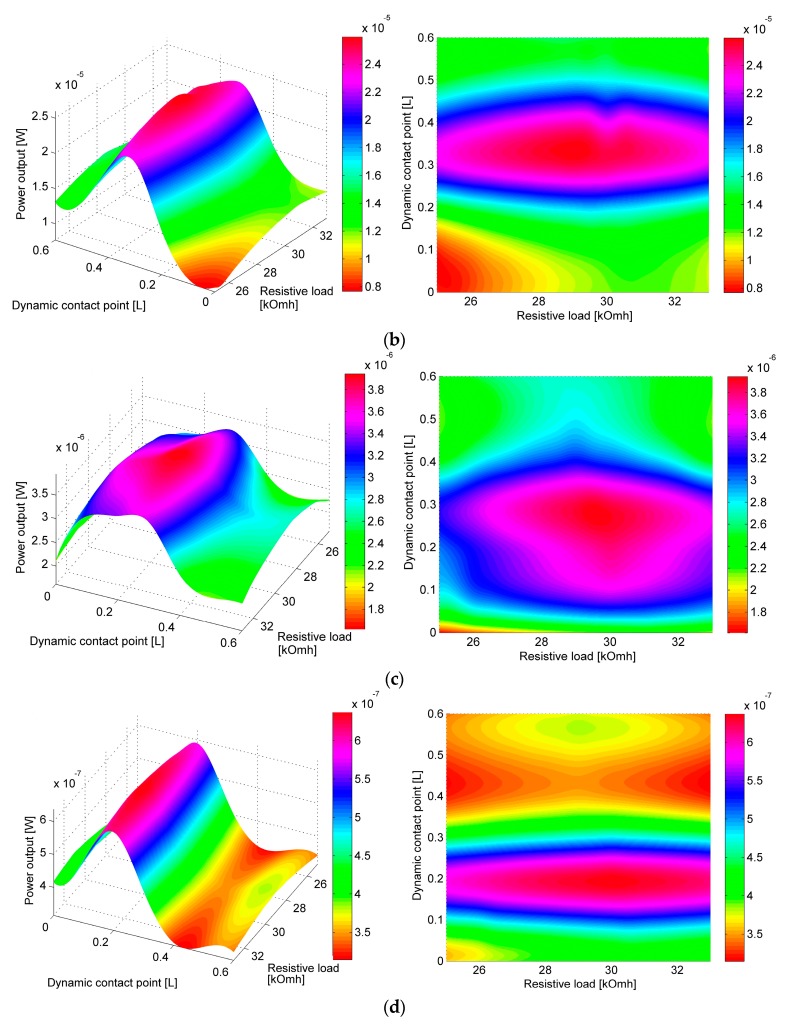

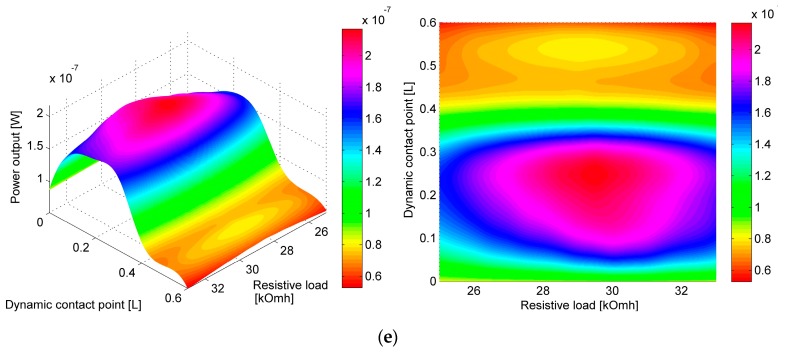
RMS Power output of PVEH with different resistive loads and dynamic contact point position. Isometric and top view with natural frequency ratio $\omega_{1}^{PVEH}/\omega_{1}^{LFR}=$ (**a**) 8; (**b**) 6; (**c**) 4; (**d**) 3; (**e**) 2.

From 10 to 12 PVEH power output curves with different resistive loads $R_{L}$ connected for each LFR-PVEH tandem configuration and impact point were obtained. The optimal resistive load for all setups was obtained from steady state analysis and was found to vary between 28 kΩ and 31 kΩ. The results were obtained by a trial and error method, i.e., by investigating the dynamic responses of the LFR-PVEH tandem with different loads $R_{L}$ connected. The figures illustrating the rms power output at different contact point positions and varying resistive load *R_L_* can be seen in Figure 5a–e. The results clearly depict that one can get maximum power output if the contact is located at 0.2 L to 0.3 L from the tip of PVEH. The size of optimal resistance varies with varying contact point position, i.e., a larger resistive load is necessary if the contact point is located closer to the free end of the cantilever.

The values of resistive loads at which maximum RMS power output was achieved are listed in Table 3, and the actual maximum RMS power output values for each configuration are given in Table 4. The trend is clear, and it shows that higher resistive load $R_{L}$ is needed closer to the free end. From Table 3 and Figure 5a–e it can be seen that the optimal resistive load $R_{L}$ has highest values when the contact point is at the free end of PVEH (0 L); as the contact point shifts by 0.6 L towards the fixed end, the size of optimal resistive load gradually decreases by ~7%. This is true for all investigated natural frequency ratios. Since for each investigated tandem configuration with the same natural frequency ratio the gap distance between the two cantilevers and the excitation parameters is kept constant, a conclusion can be drawn that only a change in the contact location can account for a change in the size of optimal resistive load and maximum RMS power output of each tandem configuration.

The difference of optimal resistive load size for the same contact point at different natural frequency ratios is comparatively small (~1.4%). In Table 4 the RMS power output of different LFR-PVEH tandem configurations at different contact points with optimal resistive load values are given. The data show that the maximum RMS power output is achieved if the contact point is located in the interval from 0.2 L to 0.3 L from the tip of PVEH. Data also show that for lower LFR $\omega_{1}^{LFR}$ frequencies the power output is higher than for corresponding impact point locations at higher $\omega_{1}^{LFR}$ frequencies. From Table 4 it was calculated that for two-fold natural frequency ratio decrease, the power output increases four to seven times.

**Figure 6:**
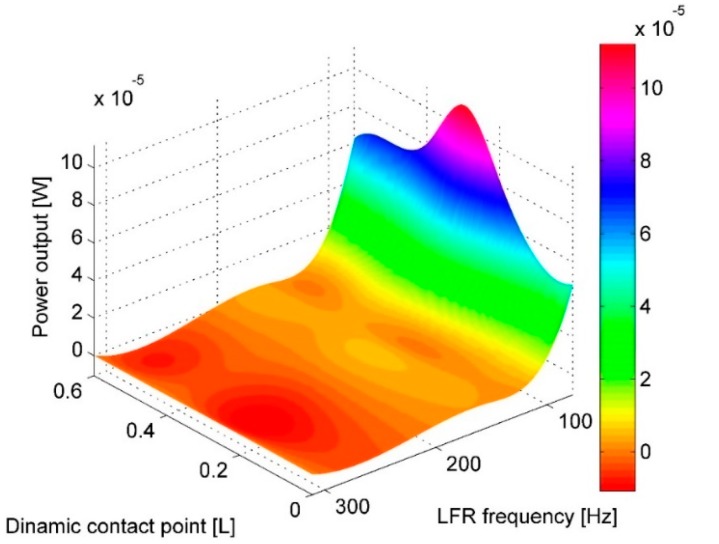
RMS power output of different tandem configurations and contact points with optimal resistive load *R_L_*.

From Table 4 it can be seen that the contact point at which the maximum RMS power output is obtained is slightly shifting towards the free end of PVEH, for natural frequency ratio $\frac{\omega_{1}^{PVEH}}{\omega_{1}^{LFR}}=$ 2 maximum power output is obtained at 0.2 L, while for higher natural frequency ratios $\omega_{1}^{PVEH}/\omega_{1}^{LFR}$ maximal power output was obtained at 0.3 L. A conclusion can be drawn that for lower natural frequency ratios, the contact point shall also be located closer to the free end of PVEH. Figure 6 illustrates the data given in Table 4.

To compare power outputs obtained at different base excitation frequencies of LFR, an efficiency criterion was invented:

(11) $$P_{eff}=\omega_{1}^{i}\times P_{n}^{i}$$

where $P_{n}^{i}$ is RMS power output at the *i*th ratio of frequencies and the nth position of the impact point at PVEH, the obtained *P_eff_* values are given in Table 5. It can be seen that a greater output is still obtained from the tandem with higher natural frequency ratio. It can still be seen that power output from tandem’s configuration with higher natural frequency ratio $\omega_{1}^{PVEH}/\omega_{1}^{LFR}$ is higher.

The difference in frequency only partially accounts for the significant difference between maximal RMS power output of harvester tandems with different normal frequency ratios $\omega_{1}^{PVEH}/\omega_{1}^{LFR}$. After the normalization, the difference between maximal RMS power outputs of tandem configurations with frequency output ratios $\omega_{1}^{PVEH}/\omega_{1}^{LFR}\;$ = 8 and 6 at 0.3 L is 306% (difference of 410% before normalization), while for $\omega_{1}^{PVEH}/\omega_{1}^{LFR}$ = 6 and 4 at 0.3 L the difference is 402% (605% before normalization). This is true for all investigated natural frequency ratios.

**Figure 7:**
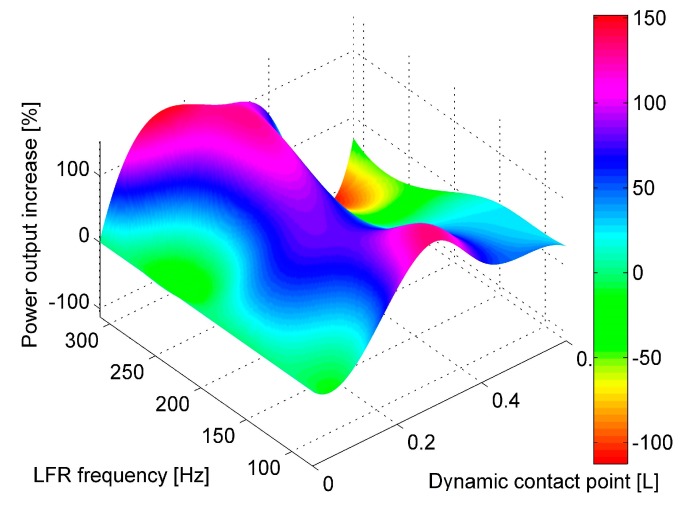
Comparison of RMS power output at different contact points with respect to PVEH power output when dynamic contact point is at 0 L.

**Figure 8:**
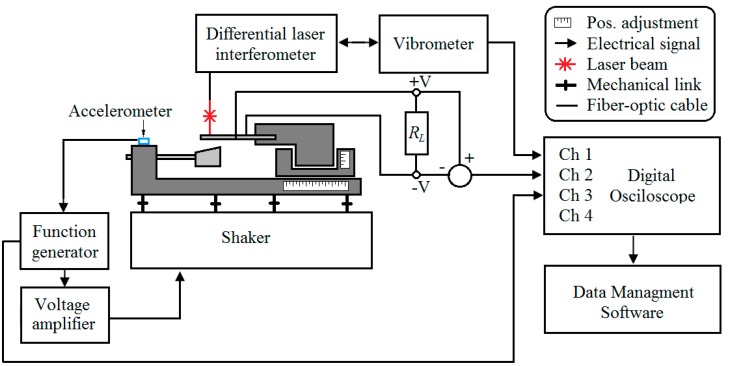
Schematic representation of experimental setup.

To compare the effect of shifting a dynamic contact point along the length of the cantilever, a comparison of RMS power output at different positions of dynamic contact points to RMS power output at 0 L position was performed. The results are presented in Table 6 and Figure 7, with values expressed as percentage difference of RMS power output at a certain contact point in comparison to power output at 0 L point. The results are also presented graphically in Figure 8. Results dictate that for a lower natural frequency ratio (or higher base excitation and LFR natural frequency) the point of maximum power output drifts slightly towards the free end of the cantilever (0 L).

It can also be observed that for natural frequency ratio $\omega_{1}^{PVEH}/\omega_{1}^{LFR}$ 6 and 4, higher power outputs are obtained when the contact point is located from 0.2 L and towards the fixed end of PVEH if compared to power output when the contact point is located at the tip of PVEH. In fact, for the mentioned tandem configurations, the power output from impact at 0 L is the lowest (power output obtained from impacting at 0.6 L still produces from 20% to 30% higher power output if compared to 0 L). For frequency ratio $\omega_{1}^{PVEH}/\omega_{1}^{LFR}$ 3 and 2, the power output closer to the fixed end (0.5 L and 0.6 L) already produces lower power output if compared to power output at 0 L. This tendency can be related to the amount of kinetic energy supplied by the LFR necessary to deflect the PVEH.

The tendency is clear here, and it suggests that the optimal contact point for different natural frequency ratios is always around 0.2 L to 0.3 L, and the increase in power output achieved there is significant. This can be related to the second transverse vibration mode shape which can be induced if the impact is located at 0.216 L from the tip, because at this position the nodal point of the second transversal vibration mode form exists.

## 4. Experimental Verification of FEM Model

For experimental verification of the derived FEM model of the frequency-up converting tandem, a prototype system was fabricated. Its schematic representation can be seen in Figure 8.

The low frequency cantilever was built from steel with a proof mass attached to its tip, whereas the high frequency harvester was built from stainless steel with a bulk PZT-5H sheet glued on top. Dry adhesives were used for bonding both layers. The displacements were measured using a Doppler Vibrometer (OFV-512 differential laser interferometer, Polytec, Waldbronn, Germany) with a Polytec OFV-5000 controller (Polytec, Waldbronn, Germany) connected to it. The low frequency generator was excited by an electromagnetic shaker. The signal was controlled by a 33220A function generator (Keysight, Santa Clara, CA, USA), and the VPA2100MN voltage amplifier (HQ Power, Gavere, Belgium) was used to amplify the signal. A single axis accelerometer was attached to the acrylic glass support mounted on top of the shaker to measure the excitation amplitude (single axis accelerometer KS-93, sensitivity—0.35 mV/(m/s^2^). The readings were taken by a 3425 USB oscilloscope. The LFR was clamped to the test rig in a fixed position while the PVEH was mounted onto a movable structure which allowed changing the gap distance and the contact point between LFR and PVEH. The whole structure was mounted on top of a shaker. The cantilevers were manufactured using laser cutting.

The first experiment was done to compare the dynamic response of PVEH under contact excitation under open circuit conditions (RL = 10 MΩ). The device was subjected to a 77 Hz base excitation which induced a ~622 Hz frequency response via impact coupling in the high frequency harvester due to frequency up-conversion phenomena.

**Figure 9:**
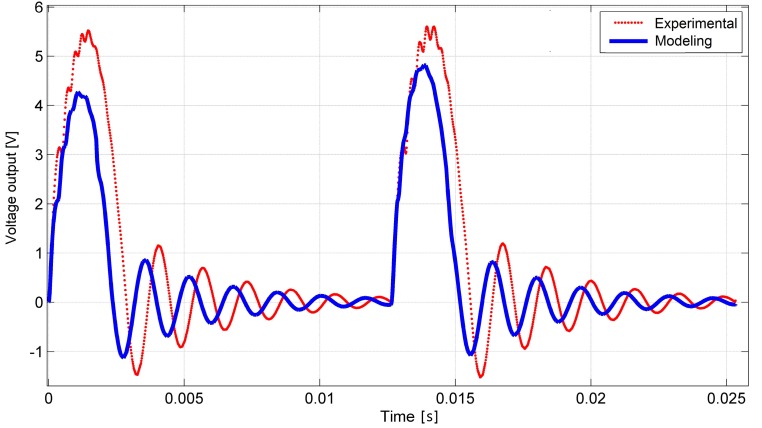
Experimental vs. modeling obtained open circuit voltage output of PVEH under dynamic excitation by 77 Hz LFR at 0.2 L dynamic impact contact point.

With these parameters, a voltage-time dependence was obtained experimentally, which, together with the modeling results, is shown in Figure 9. In this figure, a transient process taken from steady-state vibrations is shown. The difference between experimentally and modeling obtained voltage outputs from the impact is around 15%. From the experimental curve, it can also be seen that during the impact higher transverse vibration modes are induced in PVEH. The duration of impact was a little longer in the experimental mode. This can be explained by the difference in LFR frequency or damping parameters versus the model. Despite the transient process, its duration between impacts and overall behavior show good agreement with the theoretical model.

**Figure 10:**
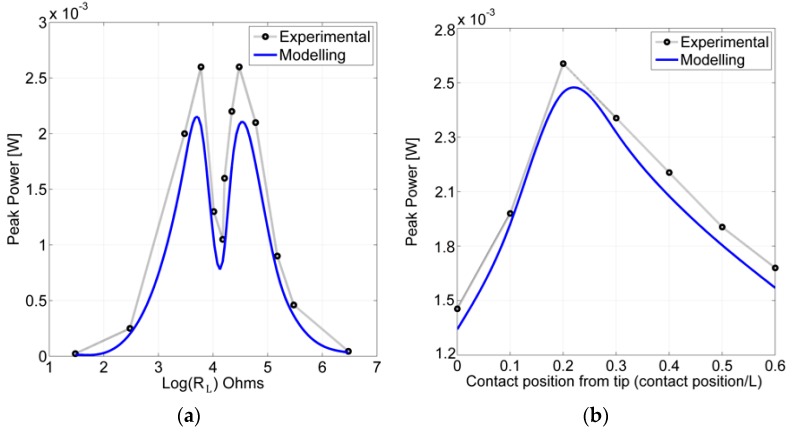
Experimental and modeling maximum of harvested power output as a function of: (**a**) load resistance under sinusoidal and impact excitation, contact position—0.2 L, natural frequency ratio $\omega_{1}^{PVEH}$/$\omega_{1}^{LFR}=8$; (**b**) contact position at natural frequency ratio $\omega_{1}^{PVEH}$/$\omega_{1}^{LFR}=8$, resistive load attached *R_L_* = 29.5 kΩ.

Figure 10a presents a comparison of modeling and experimentally obtained peak of harvested power as a function of load resistance under highly nonlinear vibro-shock inputs to the transducer. The experiment was done using these parameter values: contact position—0.2 L, natural frequency ratio $\omega_{1}^{PVEH}$/$\omega_{1}^{LFR}=8$. The resistive load values in horizontal axis are shown on a logarithmic scale, thus two peaks in the experimental curve can be translated to ~6.5 kΩ for the first peak and ~31 kΩ for the second peak. The modeling results show good agreement with experimental the results. This clear gap in peak power output as a function of resistive load could be explained by sinusoidal and impact driven vibrations. Results show that experimentally obtained values were approximately 15% higher than the results obtained from modelling.

Relationships between the output power and the contact point position are presented in Figure 10b where experimental and modelling results of peak harvested power as a function of a contact position are compared. From this relationship, in both the experimental and modeling case the peak power output was obtained at the 0.2 L contact position.

The experiment was done using these parameter values: natural frequency ratio $\omega_{1}^{PVEH}$/$\omega_{1}^{LFR}=8$, resistive load attached *R_L_* = 29.5 kΩ. Experimental and modelling results are in a good agreement, and the experimental results show the model underpredicted the power output by ~8%. The peak power output presented in Figure 10b also shows a good agreement with the RMS power output at different contact positions presented in Table 4 and Figure 5 where the highest calculated RMS power output was found to be at 0.2 L–0.3 L. Since the modeling and experimental results display satisfactory agreement, the model is assumed to be correct.

## 5. Conclusions

Mathematical and finite element models of a multi-beam vibro-shock energy harvester were created, which were realized using Comsol Multi-physics software. The validation of the PVEH peak power output between the physical vibro-shock harvesting system and the mathematical model was done and good agreement of the results was achieved. Experimental and simulated optimal resistive loads were found by the method of trial and error from the vibro-shock system dynamic analysis. The resistive load RL has two distinct optimal values, one about 6.5 kΩ and second was found to be from 28.5 kΩ to 31 kΩ and depends on the location of the contact point. Higher resistive loads are needed if the contact point is located closer to the tip of the cantilever (0 L).

By simulation and DFT analysis results it was found that a higher frequency ratio between the LFR and PVEH provides higher power output. From the frequency-normalized data the maximal power output is obtained when frequency ratio $\omega_{1}^{P}$/$\omega_{1}^{LF}$ = 8 ($\omega_{1}^{LFR}=77\;\mathrm{Hz}$), while other ratios produced 3 to 16 times less power. It was shown that the maximal RMS power is achieved when a contact point is located at 0.2 L to 0.3 L distance from the PVEH tip. These modelling results were confirmed by experimental results where the relationship between the output peak power and the contact point position was investigated. Experimental and modelling results are in a good agreement, and the experimental results show the model underpredicted the power output by ~8%. This is related to the induction of the higher vibration modes which, as a result, increase the amount of generated energy.

## Figures and Tables

**Table 1:** Mechanical and geometrical properties of the proposed piezoelectric vibration energy harvester.

Parameter	Value
$\omega_{1}^{PVEH},\;\mathrm{Hz}$	622
Density ρ, kg/m^3^	1430
Young’s modulus, Pa	4.6 × 10^9^
Poisson’s ratio	0.33
Length *l*, m	0.01
Width *a*, m	0.003
Thickness *b*, m	0.0001

**Table 2:** PZT-5H properties.

Parameter	Value
Piezoelectric strain constant d_31_, (pC/N)	23
Piezoelectric stress constant g_31_, (10^−3^ Vm/N)	216
Electromechanical coupling factor k_t_	12%
Capacitance C, nF	1.4–2.8
Young’s modulus Y, 10^9^ N/m^2^	4
Permittivity ε, 10^−12^ F/m	110
Mass Density ρ, kg/m^3^	1780
Thickness t, m	0.00008

**Table 3:** Optimal resistive [kΩ] loads for different LRF/PVEH tandem configuration.

	$\frac{\omega_{1}^{PVEH}}{\omega_{1}^{LFR}}$	Dynamic Contact Point Position from the Tip of the PVEH Cantilever						
		0 L	0.1 L	0.2 L	0.3 L	0.4 L	0.5 L	0.6 L
**Optimal resistive load *R_L_*, [kΩ]**	**8**	31	30.5	29.5	29.5	29	29	29
	**6**	31	30.5	29.5	29	29	29	28.5
	**4**	31	30	30	29.5	29	29	28.5
	**3**	31	30.5	30	30	29	29	28.5
	**2**	30.5	30	29.5	29.5	29	29	28.5

**Table 4:** RMS power output [W] of different tandem configurations at optimal resistive loads.

	$\frac{\omega_{1}^{PVEH}}{\omega_{1}^{LFR}}$	Dynamic Contact Point Distance from the Tip of the Cantilever						
		0 L	0.1 L	0.2 L	0.3 L	0.4 L	0.5 L	0.6 L
**PVEH RMS Power output, (W)**	**8**	4.81 × 10^−5^	5.11 × 10^−5^	9.20 × 10^−5^	1.12 × 10^−4^	8.11 × 10^−5^	7.09 × 10^−5^	6.25 × 10^−5^
	**6**	1.22 × 10^−5^	1.31 × 10^−5^	2.32 × 10^−5^	2.73 × 10^−5^	2.19 × 10^−5^	1.71 × 10^−5^	1.53 × 10^−5^
	**4**	2.25 × 10^−6^	3.51 × 10^−6^	4.21 × 10^−6^	4.51 × 10^−6^	3.19 × 10^−6^	2.81 × 10^−6^	2.73 × 10^−6^
	**3**	4.31 × 10^−7^	5.52 × 10^−7^	7.96 × 10^−7^	8.71 × 10^−7^	4.93 × 10^−7^	3.65 × 10^−7^	3.75 × 10^−7^
	**2**	7.08 × 10^−8^	1.12 × 10^−7^	1.53 × 10^−7^	1.35 × 10^−7^	9.30 × 10^−8^	7.04 × 10^−8^	4.78 × 10^−8^

**Table 5:** Frequency normalized RMS power output [W] for different tandem configurations at optimal resistive loads.

	$\frac{\omega_{1}^{PVEH}}{\omega_{1}^{LFR}}$	Dynamic Contact Point Distance from the Tip of the Cantilever						
		0 L	0.1 L	0.2 L	0.3 L	0.4 L	0.5 L	0.6 L
**Normalized PVEH RMS Power output**	**8**	3.70 × 10^−3^	3.93 × 10^−3^	7.08 × 10^−3^	8.62 × 10^−3^	6.24 × 10^−3^	5.46 × 10^−3^	4.81 × 10^−3^
	**6**	1.26 × 10^−3^	1.35 × 10^−3^	2.39 × 10^−3^	2.81 × 10^−3^	2.26 × 10^−3^	1.76 × 10^−3^	1.58 × 10^−3^
	**4**	3.49 × 10^−4^	5.44 × 10^−4^	6.53 × 10^−4^	6.99 × 10^−4^	4.94 × 10^−4^	4.36 × 10^−4^	4.23 × 10^−4^
	**3**	8.92 × 10^−5^	1.14 × 10^−4^	1.65 × 10^−4^	1.80 × 10^−4^	1.02 × 10^−4^	7.56 × 10^−5^	7.76 × 10^−5^
	**2**	2.20 × 10^−5^	3.48 × 10^−5^	4.76 × 10^−5^	4.20 × 10^−5^	2.89 × 10^−5^	2.19 × 10^−5^	1.49 × 10^−5^

**Table 6:** Comparison between tandem power output at different positions of contact point and a contact position at the tip.

	$\frac{\omega_{1}^{PVEH}}{\omega_{1}^{LFR}}$	Dynamic Contact Point Distance from the Tip of the Cantilever						
		0 L	0.1 L	0.2 L	0.3 L	0.4 L	0.5 L	0.6 L
**RMS Power output, (%)**	**8**	0	6.21%	91.27%	132.85%	68.61%	47.40%	29.94%
	**6**	0	7.30%	90.16%	123.77%	79.51%	40.16%	25.41%
	**4**	0	56.02%	87.11%	100.44%	41.78%	24.89%	21.33%
	**3**	0	28.16%	84.69%	102.09%	14.39%	−15.31%	−12.99%
	**2**	0	58.29%	116.10%	90.82%	31.36%	−0.56%	−32.49%
